# Universal disease biomarker: can a fixed set of blood microRNAs diagnose multiple diseases?

**DOI:** 10.1186/1756-0500-7-581

**Published:** 2014-08-30

**Authors:** Y-h Taguchi, Yoshiki Murakami

**Affiliations:** Department of Physics, Chuo University, 1-13-27 Kasuga, Bunkyo-ku, 112-8551 Tokyo, Japan; Department of Hepatology, Osaka City University, Graduate School of Medicine, 1-4-3 Asahimachi, Abeno-ku, 545-8585 Osaka, Japan

**Keywords:** Disease biomarker, Universality, Blood microRNA

## Abstract

**Background:**

The selection of disease biomarkers is often difficult because of their unstable identification, i.e., the selection of biomarkers is heavily dependent upon the set of samples analyzed and the use of independent sets of samples often results in a completely different set of biomarkers being identified. However, if a fixed set of disease biomarkers could be identified for the diagnosis of multiple diseases, the difficulties of biomarker selection could be reduced.

**Results:**

In this study, the previously identified universal disease biomarker (UDB) consisting of blood miRNAs that could discriminate between patients with multiple diseases and healthy controls was extended to the recently reported independent measurements of blood microRNAs (miRNAs). The performance achieved by UDB in an independent set of samples was competitive with performances achieved with biomarkers selected using lasso, a standard, heavily sample-dependent procedure. Furthermore, the development of stable feature extraction was suggested to be a key factor in constructing more efficient and stable (i.e., sample- and disease-independent) UDBs.

**Conclusions:**

The previously proposed UDB was successfully extended to an additional seven diseases and is expected to be useful for the diagnosis of other diseases.

**Electronic supplementary material:**

The online version of this article (doi:10.1186/1756-0500-7-581) contains supplementary material, which is available to authorized users.

## Background

Identification of biomarkers is important for the diagnosis of disease. By using biomarkers with high specificity for certain diseases, patients can be identified without diagnosis by doctors. After diagnosis using biomarkers, it is hoped that fewer patients will require diagnosis by a doctor. This enables doctors to diagnose a limited number of screened patients in more detail. Blood is a useful source of biomarkers. Numerous compounds/proteins in blood have been identified as effective biomarkers that allow the early diagnosis of several diseases (e.g., [1–3]). One disadvantage of this system is that distinct compounds/proteins are required to diagnose individual diseases, because diagnoses are usually based on the observation of unexpected values of compounds/proteins. When following this strategy, new compounds/proteins that increase or decrease in specific diseases should be identified. This system of biomarker identification incurs high costs because of the measurements of each biomarker. Thus, it is difficult to test for many diseases simultaneously because the number of diseases tested is proportional to the cost. The identification of a universal disease biomarker (UDB) that can diagnose multiple diseases simultaneously would be useful and economically beneficial. However, identifying a UDB using the traditional strategy of one compound/protein for one disease is unlikely.

Despite this difficulty, several studies have attempted to identify UDBs. For example, interleukin-8 (IL-8) was thought to be a UDB [4] as it was reported to be a useful biomarker for multiple diseases including urinary bladder cancer, prostatitis, acute pyelonephritis, vesicoureteral reflux, pulmonary infections, osteomyelitis, inflammatory bowel disease, chorioamnionitis, nosocomial bacterial infections, and non-Hodgkin’s lymphoma. Despite the apparent usefulness of IL-8 as a UDB, it has a strong tendency to increase non-specifically in individuals because most inflammatory conditions induce its production, therefore it might be considered together with other biomarkers. Another UDB is pHLIP and acidity, which although limited to cancer diagnosis was proposed to be a UDB for cancers [5]. Fendos and Engelman successfully and noninvasively labeled tumor tissues using a pH-sensitive biosensor. pHLIP also labeled tumors independent of the type of cancer. Another example of a UDB is FibroTest [6], which was used to diagnose several liver diseases including alcoholic liver disease, Hepatitis B virus, Hepatitis C virus, and Nonalcoholic fatty liver disease. FibroTest consists of a six-parameter blood test, *α*2-macroglobulin, Haptoglobin, Apolipoprotein A1, *γ*-glutamyl transpeptidase, Total bilirubin, and Alanine transaminase, combined with the age and gender of the patient. However, these biomarkers lacked either specificity (IL-8 is used in combination with other biomarkers for accurate diagnoses) or universality (pHLIP is used only for cancer diagnosis while FibroTest is only used to diagnose liver diseases). An ideal disease UDB should have the ability to diagnose multiple diseases compared with normal healthy controls. One method to achieve this is by the combination of multiple biomarkers, as used for the FibroTest. Although FibroTest has fixed coefficients to construct a UDB, if varying coupling constants allows the diagnosis of distinct multiple diseases, biomarkers that consist of multiple individual biomarkers have the potential to be UDBs.

Recently, blood microRNAs (miRNA) have been identified as promising disease biomarkers [7]; combinations of mir-498 clusters are potential biomarkers for pregnancy, although pregnancy is not a disease. Blood miRNAs were also identified as anti-doping biomarkers [8], biomarkers of peripheral arterial disease [9], acute myocardial infarction and underlying coronary artery stenosis [10], and acute graft-versus-host disease [11]. They are also stable biomarkers [12]. Furthermore, although combinatorial circulating biomarkers are considered potential effective biomarkers for various diseases [13–20], combinations for the diagnosis of individual diseases often fluctuate between studies. For example, two recent distinct studies that tried to construct combinatorial blood miRNA biomarkers for the diagnosis of Alzheimer’s disease had no common miRNAs [21, 22]. Even for the diagnosis of an individual disease, there is often no unique combination of blood miRNAs. This suggests that a UDB is unlikely to be constructed from multiple blood miRNAs.

In contrast to these studies, we recently identified a potential UDB consisting of blood miRNAs [23]. Ten to 12 common blood miRNAs could be used to diagnose 13 various diseases from normal controls. Although this demonstrated the potential of blood miRNAs to be used as a UDB, the study used samples taken from only one study with shared normal controls. Thus, further studies are required to provide convincing data. In the current study, we cross-validated the previously proposed UDB [23] of 12 fixed miRNAs by investigating whether miRNAs could diagnose an additional seven distinct diseases using blood miRNAs that were recently reported and were not available when the previous study [23] was performed. The discriminatory ability of a UDB composed of 12 fixed blood miRNAs was competitive compared with that using a conventional method and miRNAs selected by a recently proposed principal component analysis (PCA)-based unsupervised feature selection method [23].

## Results and discussion

### Universality of UDB

To determine whether previously identified UDBs consisting of blood miRNAs [23] were universal, we evaluated their performance using seven independent data sets targeting seven diseases (see Methods). Although 10 miRNAs were selected for each disease from a total of 13 diseases in the previous study, 12 combined blood miRNAs (hsa-miR-425, hsa-miR-15b, hsa-miR-185, hsa-miR-92a, hsa-miR-140-3p, hsa-miR-320a, hsa-miR-486-5p, hsa-miR-16, hsa-miR-191, hsa-miR-106b, hsa-miR-19b, and hsa-miR-30d) were used to form the UDB in this study. Missing miRNAs in the data sets were excluded from the discrimination.

**Figure 1:**
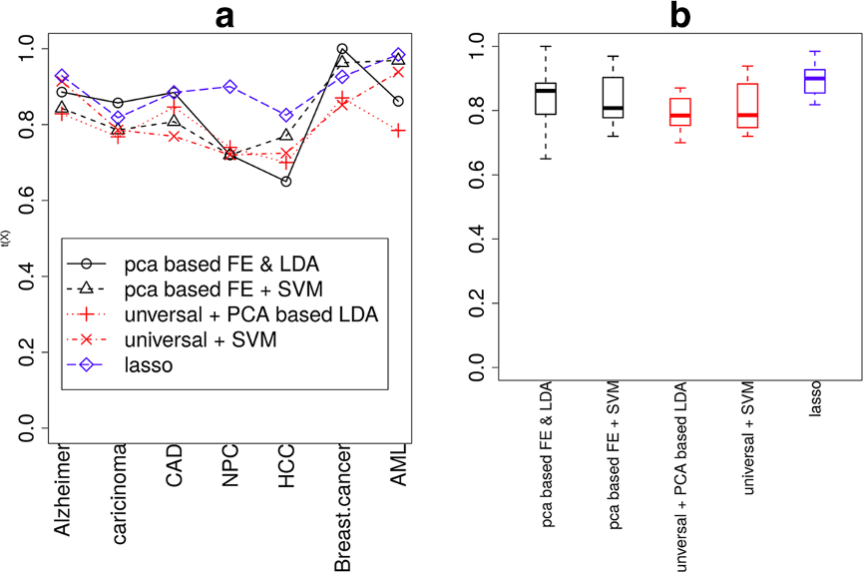
**Accuracies achieved by various discrimination methods and FEs.** **(a)** Dependence upon diseases and methods. **(b)** Boxplot of accuracies.

In Figure 1, the accuracy achieved by PCA-based liner discriminant analysis (LDA, red crosses) and support vector machine (SVM, red x-marks) using UDB is shown (also red boxes in Figure 1(b)). Mean accuracies were 0.791 and 0.815, respectively, and they were coincident with the mean accuracy (0.784) estimated using PCA-based LDA with UDB in a previous study [23] (see Table 1). Values of accuracy together with sensitivity and specificity values are also listed in Table 1. It was observed that performances were independent of the methods and samples, demonstrating the usefulness of the UDB. More detailed performances and their evaluations, i.e., true and false positives and negatives in a 2×2 tables together with *P*-values computed by Fisher’s exact test, odds ratio and area under the receiver operating characteristic (ROC) area under the curve (AUC), are shown in Additional file 1: Table S2.

**Table 1:** **Performance of UDB with PCA-based LDA and SVM**

Diseases	Accuracy	Sensitivity	Specificity
PCA-based LDA			
AD	0.829	0.833	0.818
Carcinoma	0.768	0.730	0.800
CAD	0.846	0.846	0.846
NPC	0.740	0.806	0.632
HCC	0.700	0.700	0.700
BC	0.870	0.813	0.955
AML	0.784	0.769	0.846
Mean	0.791	0.785	0.800
Mean of previous study [23]	0.784	0.750	0.800
SVM			
AD	0.914	0.917	0.909
Carcinoma	0.786	0.867	0.692
CAD	0.769	0.769	0.769
NPC	0.720	0.806	0.579
HCC	0.725	0.550	0.900
BC	0.852	0.813	0.909
AML	0.938	0.981	0.769
Mean	0.815	0.815	0.800

AD, Alzheimer’s disease; CAD, coronary artery disease; NPC, nasopharyngeal carcinoma; HCC, hepatocellular carcinoma; BC, breast cancer; AML, acute myeloid leukemia; UDB, universal disease biomarker; SVM, support vector machine; LDA, linear discriminant analysis; PCA, principal component analysis. Data from previous study [23] are also shown for comparison.

### Comparison of performances between UDB and lasso

Although Table 1 shows the usefulness of a UDB consisting of blood miRNAs, it is important to determine how effective the UDB is when compared with conventional methods (i.e., non-universal, sample-dependent sets). We performed lasso-based discrimination (see Methods) between healthy controls and patients of each disease. Lasso-based discrimination was used so that performances of feature extraction (FE) between unsupervised FE and lasso could be compared. In addition, there are generally limited numbers of individual miRNAs that exhibit significant differences between normal controls and patients (see below), thus selection based on significant differences between patients and healthy controls as usual was difficult. The results are shown in Table 2 and Figure 1 (blue diamonds and a blue box in Boxplot). More detailed performances and their evaluations, i.e., true and false positives and negatives in a 2×2 tables of lassobased discrimination together with *P*-values computed by Fisher’s exact test, odds ratios and AUC, are shown in Additional file 1: Table S3. Although performances achieved by lasso-based discrimination were better than by PCA-based LDA with UDB (Table 1), those achieved by SVM with UDB were not significantly lower than the lasso-based discrimination (although three tests were performed, *t*-test, Wilcoxon rank sum test and Kolmogorov-Smirnov test, no *P*-values lower than 0.05 were detected). Since the lack of significance was because of large fluctuations in performances achieved by SVM with UDB, this suggested UDB might not be as effective as lasso-based discrimination. However, the possibility that UDB is as effective as standard discrimination using sample-dependent (not universal) features is indicated.

**Table 2:** **Performance of lasso-based discrimination**

Diseases	Accuracy	Sensitivity	Specificity	Optimal***s***
AD	0.928	0.979	0.818	0.09
Carcinoma	0.818	0.867	0.760	0.9
CAD	0.884	0.769	1.000	0.24
NPC	0.900	0.935	0.842	1
HCC	0.825	0.650	1.000	0.03
BC	0.925	0.906	0.955	0.46
AML	0.985	1.000	0.923	0.64
Mean	0.895	0.872	0.900	

AD, Alzheimer’s disease; CAD, coronary artery disease; NPC, nasopharyngeal carcinoma; HCC, hepatocellular carcinoma; BC, breast cancer; AML, acute myeloid leukemia. *s* (fraction) is used for the predict.lars function (see Methods).

### Stability of FE: the condition to get UDB

To understand why we could successfully identified a UDB in the previous study that could never be indeitified by anyone, the stabilities of FE were compared between lasso and PCA-based unsupervised FE. PCA-based unsupervised FE was used for the previous UDB discovery [23]. The importance of stability was previously demonstrated by Wehrens *et al.* [24], who showed that a stable FE improved the performance.

**Figure 2:**
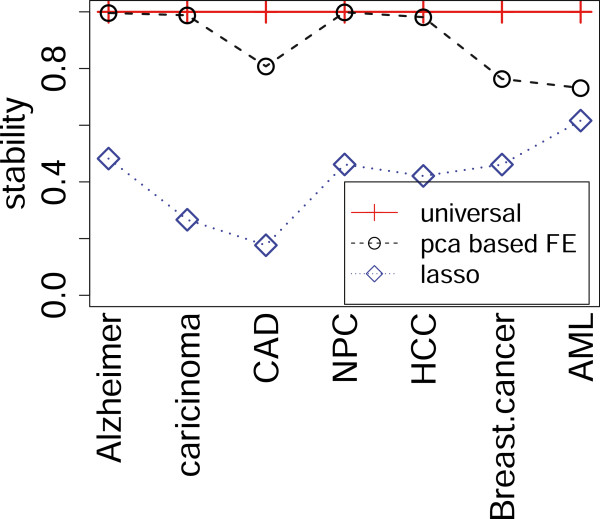
**Stabilities achieved by UDB, PCA-based FE and lasso.** Since no selections are required for UDB, stabilities of UDB are uniquely designated as 1.

Figure 2 shows the stabilities *S* (see Methods) of lasso-based discrimination (blue diamonds). Generally, the stabilities were very low and each miRNA was selected as a biomarker at most for half the trials. Thus, lasso does not have the ability to provide UDBs, because it could not select stable (sample-independent) biomarkers for each disease. One may suppose that the stabilities will improve if miRNAs that exhibit significant differences between healthy controls and patients are identified and selected. However, this is not currently a realistic strategy, since there are insufficient numbers of miRNAs (often<10) that exhibit significant differences between healthy controls and patients (Table 3). For coronary artery disease (CAD) and hepatocellular carcinoma (HCC), no miRNAs have been identified that exhibit significant differences between normal controls and patients in the present data sets.

**Table 3:** **The number of miRNAs that exhibit significant differences between normal controls and patients for each disease**

Diseases	Significant	Not significant
AD	4	498
Carcinoma	7	558
CAD	0	746
NPC	264	622
HCC	0	255
BC	86	188
AML	6	122

AD, Alzheimer’s disease; CAD, coronary artery disease; NPC, nasopharyngeal carcinoma; HCC, hepatocellular carcinoma; BC, breast cancer; AML, acute myeloid leukemia. For more details, see Methods.

However, PCA-based unsupervised FE (black circles in Figure 2) showed significantly larger *S* values than lasso. In addition, performances were comparative with those achieved by lasso (Table 4, black circles and triangles in Figure 1(a) and black box in Figure 1(b)). More detailed performances and their evaluations, i.e., true and false positives and negatives in a 2×2 tables together with *P*-values by Fisher’ exact test, odds ratio and AUC, are shown in Additional file 1: Table S4.

**Table 4:** **Performance of miRNAs selected by PCA-based FE with PCA-based LDA and SVM**

				Number of		
Diseases	Accuracy	Sens.	Spec.	miRNAs ^*^	PCs ^+^	***Δ*** ^#^
PCA-based LDA						
AD	0.886	0.917	0.818	22	16	2.5
Carcinoma	0.857	0.846	0.867	36	2	7
CAD	0.885	0.923	0.846	16	14	9
NPC	0.720	0.806	0.579	28	18	5
HCC	0.650	0.600	0.700	8	1	7
BC	1.000	1.000	1.000	18	13	6
AML	0.862	0.846	0.923	11	8	7
Mean	0.837	0.848	0.819			
Mean of previous study [23]	0.784	0.750	0.800			
SVM						
AD	0.843	0.833	0.864	22		
Carcinoma	0.786	0.807	0.767	36		
CAD	0.807	0.615	1.000	16		
NPC	0.720	0.774	0.632	28		
HCC	0.770	0.550	0.850	8		
BC	0.963	1.000	0.938	18		
AML	0.969	1.000	0.846	11		
Mean	0.837	0.797	0.842			

^*^number of miRNAs selected by PCA-based FE, ^+^optimal number of PCs estimated by LOOCV, ^#^threshold value of PCA-based FE. Data from previous study [23] are also shown for comparison. AD, Alzheimer’s disease; CAD, coronary artery disease; NPC, nasopharyngeal carcinoma; HCC, hepatocellular carcinoma; BC, breast cancer; AML, acute myeloid leukemia; UDB, universal disease biomarker; SVM, support vector machine; LDA, linear discriminant analysis; PCA, principal component analysis.

Why selected biomarkers are frequently varied between samples was attributed to the difference of data normalization. However, the results shown here indicate this might be caused by using incorrect and unstable FE methods. To obtain UDB, stable FE methods should be used [23].

The study by Wehrens *et al.* [24] used PCA-based LDA to maximize the stability of FE, whereas the current study did not require better stability, as this is automatically obtained when using PCA-based unsupervised FE. Thus, stability achieved by PCA-based unsupervised FE is expected to be more robust than feature selections by stability maximization using PCA-based LDA. Moreover, to rank features based on stability, Wehrens *et al.* [24] performed time-consuming iterative cross-validations that were not required by the PCA-based unsupervised FE. Thus, PCA-based unsupervised FE methodology is less computationally challenging than feature selections by stability maximization using PCA-based LDA.

The successful identification of UDBs [23] was possibly because of stable FE methods, which we suggest are important for developing UDBs, although the stability of FE is often overlooked. To determine more efficient UDBs, searching with efficient and stable FEs is required.

### The number of features selected by FE

Previously [23], the number of features selected by PCA-based unsupervised FE was fixed at 10, because data sets analyzed previously were taken from a single study. Previous studies used the same microarray to measure miRNA expression in multiple diseases. In contrast, data sets used in the current study were heterogeneous. They were collected from multiple studies performed by independent research groups. Measurements were not performed by a single microarray but by various methods including qPCR. The sources of samples were also heterogeneous, ranging from whole blood to serum or plasma. Thus, we varied the number of features selected by PCA-based unsupervised FE between diseases (Additional file 2: Figure S1 for two-dimensional embeddings of miRNAs used for FE).

Interestingly, the optimal number of selected features was common between lasso and PCA-based unsupervised FE (Additional file 2: Figure S2). This suggests that the number of miRNAs required to discriminate healthy controls from patients is not dependent on the methods used but on the samples. This is not surprising because many sets of miRNAs discriminate between patients and normal controls if miRNAs are not independent of each other. In addition, the stability of FE is important, otherwise selected features will vary between trials.

This study did not identify a UDB from a data set we used, but rather validated the usefulness of UDBs identified in a previous study. To identify UDBs, sample preparation and measurements must be standardized to minimize the variance between samples. This should be possible because the target is uniquely independent of blood in disease.

### Toward a mechanism-based biomarker

**Figure 3:**
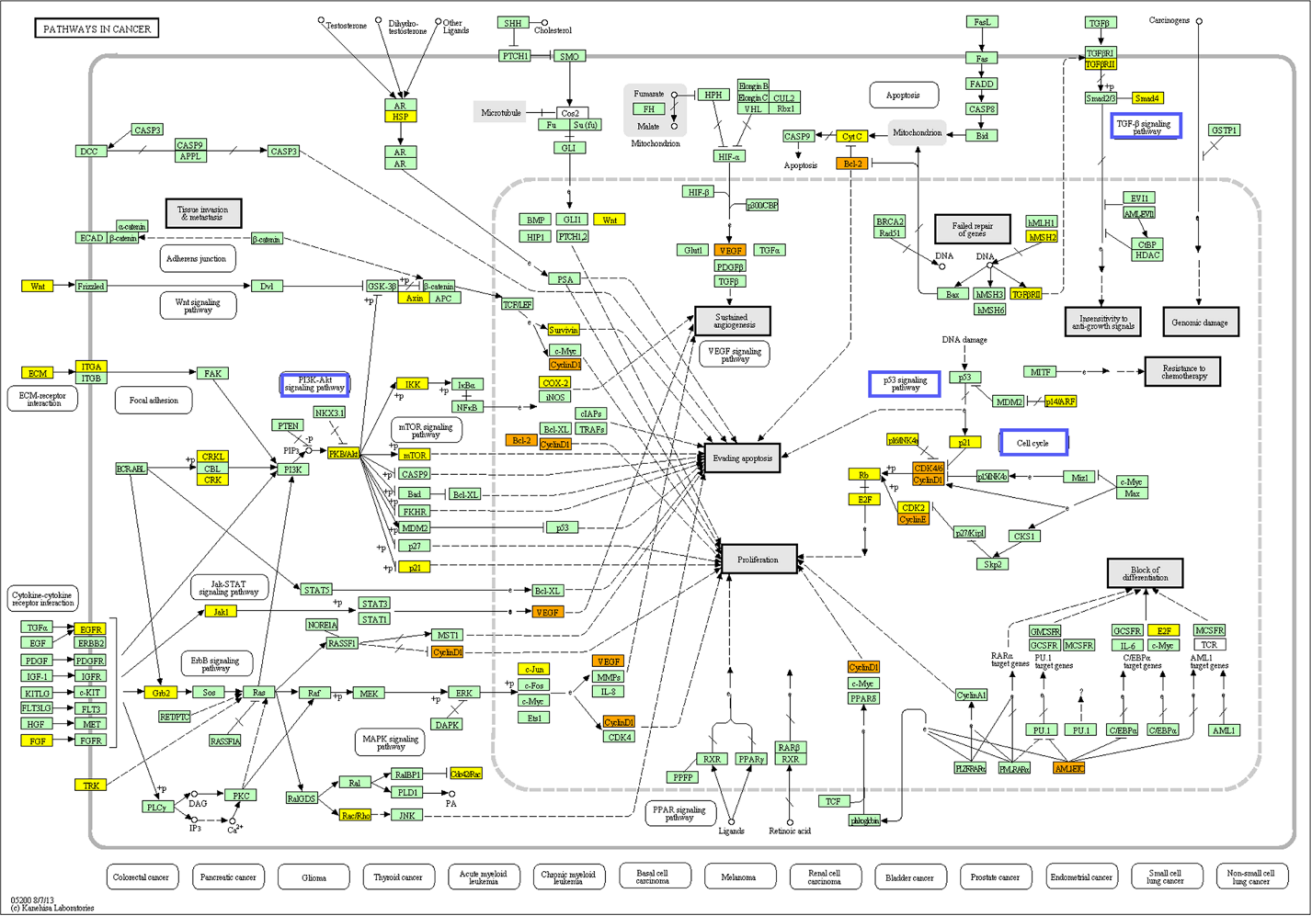
**KEGG pathway: “Pathways in cancer”.** Yellow: genes targeted by an miRNA included in the UDB in this study. Orange: genes targeted by more than one miRNAs included in the UDB in this study. Pathways surrounded by blue rectangles are listed in Table 5.

The UDB in this study was clearly decided by meta-analysis, and thus was not mechanism-based. However, if it also functions as a mechanism-based biomarker, this would be more plausible. To determine the possibility of using a UDB as a mechanism-based biomarker, we employed DIANA-mirpath [25]. Table 5 lists the 27 significant KEGG pathways reported by DIANA-mirpath (see Methods). Among 27 KEGG pathways, nine were cancer pathways (bold font). There were also five pathways (bold ilatic) that were disease pathways other than cancers. In addition, three pathways (italicized) were cancer-related pathways and four pathways (asterisked) were parts of “Pathways in cancer” (Figure 3). Thus, there were only five pathways that were not directly related to diseases. Therefore, miRNAs included in the UDB in this study were not only extensively included disease pathways, but also contributed to various disease pathways. Further experimental investigations of the expression of miRNA target genes will be required to demonstrate how UDB is involved in disease mechanisms.

**Table 5:** **KEGG pathway analysis of 12 miRNAs included in the UDB using DIANA-mirpath [** [25]**]**

	KEGG.pathway	p.value	# of genes	# of miRNAs
1	***Prion diseases***	0.00e+00	6	2
2	*Pathways in cancer*	3.00e-13	39	6
3	PI3K-Akt signaling pathway ^∗^	1.07e-11	43	4
4	TGF-beta signaling pathway ^∗^	5.98e-10	14	4
5	*Viral carcinogenesis*	1.56e-09	27	5
6	Ribosome	6.04e-09	22	1
7	**Small cell lung cancer**	6.33e-09	17	5
8	**Colorectal cancer**	1.02e-08	9	6
9	Ribosome biogenesis	2.02e-08	20	1
	in eukaryotes			
10	p53 signaling pathway ^∗^	6.75e-08	16	5
11	RNA transport	1.19e-07	28	1
12	Cell cycle ^∗^	1.56e-07	22	3
13	**Pancreatic cancer**	2.75e-07	11	5
14	***Hepatitis B***	1.17e-06	12	5
15	**Prostate cancer**	4.64e-06	16	5
16	**Bladder cancer**	6.94e-06	7	4
17	**Chronic myeloid leukemia**	2.01e-05	8	4
18	***Measles***	1.28e-04	19	5
19	Protein export	3.29e-04	9	1
20	**Non-small cell lung cancer**	3.69e-04	6	5
21	***HTLV-I infection***	1.43e-03	11	3
22	**Glioma**	1.46e-03	6	3
23	**Melanoma**	1.71e-03	7	5
24	*Transcriptional misregulation*	1.22e-02	9	3
	*in cancer*			
25	Oocyte meiosis	1.36e-02	14	1
26	Focal adhesion ^∗^	1.50e-02	8	3
27	***Epstein-Barr virus infection***	2.45e-02	19	3

Bold faces: tumors/cancers, *Bold italic*: other diseases, *Italic*: tumors/cancers related, ^*^parts of “Pathways in cancer”, and surrounded by blue rectangular in Figure 3.

### Heterogeneity of blood sources

In contrast to previous research [23] where only serum samples were used, the blood sources in this study were heterogenous, ranging from whole blood [21] to serum [26] or plasma [27] (full list of sources is shown in Additional file 1: Table S1). One may wonder why UDB works well despite this heterogeneity of sources. However, in a previous study [23], we tried to select 12 miRNAs included in UDB, not based on inference accuracy but rather by stability. That study only checked sample independency, but it is likely that sample independency is also related to source independency, since it is often as large as source dependency. miRNA expression is dependent upon both the source and patients’ age, gender, and body mass index. In addition, UDB was independent of measurement methods, i.e., NGS, microarray or qPCR (a full list of measurement methods is shown in Additional file 1: Table S1). If UDB is independent of patient properties and measurement methods, it is not surprising that UDB is also independent of sources, since all sources were taken from blood. Source independency of UDB should be investigated in more detail in the future.

### Usefulness of UDB as practical clinical tools

One may wonder if the expected accuracy (0.8) of UDB is useful or not. However, UDB can diagnose multiple diseases simultaneously. Therefore, by measuring 12 miRNAs in blood, over 20 diseases (14 diseases in the previous study [23] and seven diseases in this study) can be diagnosed. Thus, UDB can be used for pre-screening. For example, patients are diagnosed by UDB for the 20 diseases. Then, if patients are positive for one disease, further diagnosis using more precise biomarkers can confirm the diagnosis. This will be more effective and non-invasive than performing 20 independent diagnoses using disease-specific biomarkers.

## Conclusion

In this study, we demonstrated that a predefined UDB [23] could discriminate seven diseases from healthy controls. Since the diseases and samples were not included in our previous study [23] that defined UDBs, this study suggests the robustness of UDB for disease diagnosis. The performance achieved by UDB was comparative with that of lasso, the standard sample-dependent FE. Because PCA-based unsupervised FE, used for UDB identification in a previous study, outperformed lasso in terms of stability, the use of stable FE will be a key factor for discovering UDBs.

## Methods

### Blood miRNA expression profiles

Seven blood miRNA expressions used in this study were from the Gene Expression Omnibus (GEO): Alzheimer’s disease (AD) (GSE46579) [21], carcinoma (GSE37472) [26], CAD (GSE49823), nasopharyngeal carcinoma (NPC) (GSE43329), HCC (GSE50013) [27], breast cancer (BC) (GSE41922) [28] and acute myeloid leukemia (AML) (GSE49665) [29]. Detailed information is shown in Additional file 1: Table S1.

### Principal component analysis-based unsupervised feature extraction

To select blood miRNAs for the diagnosis of seven diseases, blood miRNAs were selected using the recently proposed PCA-based unsupervised FE as previously described [23, 30]. Briefly, suppose **X** is the matrix such that *x*_*ij*_ represents the amount of the *i*th miRNA expression in the *j*th sample. PCA is regarded as the eigenvalue problem


where *N* and *M* are the total number of miRNAs and samples, respectively. Here *M* is assumed to be less than *N* as is usual. *λ*_*i*_ and ***u***_*i*_ represent the eigenvalue and vector, respectively.


gives the principal component score (PCS) of *i*th miRNA. Using the obtained *x*_*ik*_,*k*=1,…,*D*(<*M*), miRNAs were determined to be embedded into low *D* dimensional space.

Multiplying **X** on both sides, the following is obtained:


where ***v***_*k*_=**X*u***_*k*_ can be regarded as an eigenvector. Then,


gives the PCS of the *j*th sample. Using the obtained *x*_*kj*_,*k*=1,…,*D*(<*M*), samples were regarded to be embedded into low *D* dimensional space.

PCA-based unsupervised FE selects outlier miRNAs in low *K*(<*M*) dimensional embedding space,


where


Typically *K* is taken to be two. Since these outliers could have a major contribution to ***u***_*k*_’s by definition, if there are a limited number of well-defined outliers, the exclusion of miRNAs other than outliers does not alter ***u***_*k*_’s. Since ***v***_*k*_ is a linear transformation of ***u***_*k*_ as shown above, the exclusion of miRNAs other than outliers does not alter ***v***_*k*_. Thus, retaining only outlier miRNAs may also preserve lower dimensional embeddings of samples that are important for disease diagnosis, e.g., discrimination between patients and healthy controls. Although this is only hypothetical, it explains why PCA-based unsupervised FE is expected to function well. Currently, there are no well-defined criteria for the selection of *Δ*. Although *Δ* was decided to include sufficient numbers (majority) of outliers, these were selected by the visual inspection of two-dimensional embedding of miRNAs. Singular decomposition-based interpretation is also available as Additional file 3: Text S1.

### Discriminatory analyses between patients and healthy controls with cross-validations

Three discriminant analyses were performed in this study as follows. The first, a PCA-based LDA, a discriminant counterpart of the partial least square (PLS), is defined as discrimination using the first *k* PCSs (i.e., from the first to the *k*th PCSs). First, PCA was applied to all samples. Then, PCA-based LDA was performed using only PCSs in the training set. Since the learning process includes unlabeled information of the test set, it is semi-supervised learning. Samples in the test set were predicted using trained PCA-based LDA. LDA was performed using lda functions in R [31] and the prediction of samples in the test set was performed by predict.lda functions in R. Optimal *k* was determined using cross-validations. The second analysis used an SVM trained with training set samples using svm function included in the e1071 R package with default settings (e.g., with the usage of Gaussian kernel), other than class.weight argument that was set to attribute equal weights to sets of normal controls and patients when the number of samples in normal controls differed from that of patients. Then, samples in the test set were predicted using predict.svm function in R. Third, lasso was used for a discrimination study. Lasso was performed using the lars function included in lars R package, attributing 1 and 2 to healthy controls and patients, respectively, and using the setting type=‘lasso’. Then, samples in the test set were predicted using predict.lars function in R for *s*=*n*/100,*n*=0,…,100 with mode=‘fraction’. Samples with predicted values larger (less) than 1.5 were regarded to be patients (healthy controls). Optimal *s* was selected by cross-validation. For all cases, leave one out cross-validation (LOOCV) was employed.

### Data normalization

Since this study is a meta-analysis using data sets collected from various independent studies employing distinct measuring methods, we normalized data sets individually by distinct methods (Table 6). Data from multiple studies were treated identically and compared. In addition, some miRNAs with abnormally large values were excluded from the analysis. Excluded miRNAs were hsa-miR-486-5p (AD), hsa-miR-223 and hsa-miR-338 (CAD), and hsa-miR-451 (NPC).

**Table 6:** **Details of data normalization**

		Data set names/	Data normalization	Datanormalization
GEO ID	Disease	Data retrieval methods	timing	methods
GSE46579	AD	GSE46579_AD_ngs_data_summarized.xls.gz	before FE	zero mean/variance is one
GSE37472	carcinoma	getGEO	before FE	zero mean/variance is one
GSE49823	CAD	getGEO	after FE	zero mean/variance is one ^∗^
GSE43329	NPC	getGEO	before FE	zero mean/variance is one ^+^
GSE50013	HCC	getGEO	before FE ^*#*^	zero mean/variance is one ^∗^
GSE41922	BC	GSE41922_series_matrix.txt.gz	after FE	zero mean/variance is one ^∗^
GSE49665	AML	getGEO	after FE	zero mean/variance is one ^∗^

^*^no normalization for SVM/lasso, ^+^no normalization for SVM with PCA-based FE, ^#^after FE for PCA-based LDA with universal features. All the sample normalizations were sample-based; i.e., each sample was normalized to have both zero mean and unit variance. AD, Alzheimer disease; CAD, coronary artery disease; NPC, nasopharyngeal carcinoma; HCC, hepatocellular carcinoma; BC, breast cancer; AML, acute myeloid leukemia. Data retrieval methods/data set names were used to name files and for analysis. getGEO indicates that individual sample profiles whose files names started with “GEO” were downloaded by the getGEO command in R.

### Stability test

On LOOCV FE, selected features (miRNAs) are listed. For lasso, miRNAs with non-zero *β*s were listed by setting type=‘coefficients’ for predict.lars function with estimated optimal *s*. Because of LOOCV, FE was performed by *M*(=the number of samples) times. Then stability was defined as


where *F*_*i*_ is the number of times that *i*th miRNA was selected within *M* times FE. Summation was performed for miRNAs that were non-zero *F*_*i*_ (i.e., selected at least once in FEs) and  is the number of miRNAs included in the summation. Larger  indicates more stable FEs.

### *P*-values computation for significant difference between healthy controls and patients

*P*-values computed for significant differences between healthy controls and patients of each disease were determined using *t*-test for each miRNA. Computed *P*-values were adjusted by BH-criterion [32] and miRNAs with adjusted *P*-values less than 0.05 were regarded to have significantly different expression between normal controls and patients.

### KEGG pathway analysis of UDB using DIANA-mirpath

DIANA-mirpath [25] was employed to investigate KEGG pathways enriched by miRNA target genes. Twelve genes were uploaded to DIANA-mirpath with the following settings: “Species” was “Human”, “FDR” correction was “yes”, “P-value threshold” was 0.05, and “Select the way to merge results” was “pathway union” (direct link to DIANA-mirpath and full list of KEGG pathways are shown in Additional file 3: Text S2 and Additional file 1: Table S5).

## Electronic supplementary material

Additional file 1: **Supporting Tables.** (XLSX 16 KB)

Additional file 2: **Supporting Figures.** (PDF 198 KB)

Additional file 3: **Supporting Texts.** (ZIP 17 KB)

## References

[1] Hanash SM, Baik CS, Kallioniemi O (2011). **Emerging molecular biomarkers–blood-based strategies to detect and monitor cancer**. Nat Rev Clin Oncol.

[2] Jellinger KA, Janetzky B, Attems J, Kienzl E (2008). **Biomarkers for early diagnosis of Alzheimer disease ’ALZheimer ASsociated gene’–a new blood biomarker?**. J Cell Mol Med.

[3] Petricoin EF, Belluco C, Araujo RP, Liotta LA (2006). **The blood peptidome: a higher dimension of information content for cancer biomarker discovery**. Nat Rev Cancer.

[4] Shahzad A, Knapp M, Lang I, Kohler G (2010). **Interleukin 8 (IL-8) - a universal biomarker?**. Int Arch Med.

[5] Fendos J, Engelman D (2012). **pHLIP and acidity as a universal biomarker for cancer**. Yale J Biol Med.

[6] Morra R, Munteanu M, Imbert-Bismut F, Messous D, Ratziu V, Poynard T (2007). **FibroMAX: towards a new universal biomarker of liver disease?**. Expert Rev Mol Diag.

[7] Williams Z, Ben-Dov IZ, Elias R, Mihailovic A, Brown M, Rosenwaks Z, Tuschl T (2013). **Comprehensive profiling of circulating microRNA via small RNA sequencing of cDNA libraries reveals biomarker potential and limitations**. Proc Natl Acad Sci USA.

[8] Leuenberger N, Robinson N, Saugy M (2013). **Circulating miRNAs: a new generation of anti-doping biomarkers**. Anal Bioanal Chem.

[9] Sluijter JP, Doevendans PA (2013). **Circulating microRNA profiles for detection of peripheral arterial disease: small new biomarkers for cardiovascular disease**. Circ Cardiovasc Genet.

[10] Wang F, Long G, Zhao C, Li H, Chaugai S, Wang Y, Chen C, Wang D. W (2013). **Plasma microRNA-133a is a new marker for both acute myocardial infarction and underlying coronary artery stenosis**. J Transl Med.

[11] Xiao B, Wang Y, Li W, Baker M, Guo J, Corbet K, Tsalik EL, Li QJ, Palmer SM, Woods CW, Li Z, Chao NJ, He YW (2013). **Plasma microRNA signature as a non-invasive biomarker for acute graft-versus-host disease**. Blood.

[12] Koberle V, Pleli T, Schmithals C, Augusto Alonso E, Haupenthal J, Bonig H, Peveling-Oberhag J, Biondi RM, Zeuzem S, Kronenberger B, Waidmann O, Piiper A (2013). **Differential stability of cell-free circulating microRNAs implications for their utilization as biomarkers**. PLoS ONE.

[13] Sheinerman KS, Umansky SR (2013). **Circulating cell-free microRNA as biomarkers for screening, diagnosis and monitoring of neurodegenerative diseases and other neurologic pathologies**. Front Cell Neurosci.

[14] Recchioni R, Marcheselli F, Olivieri F, Ricci S, Procopio AD, Antonicelli R (2013). **Conventional and novel diagnostic biomarkers of acute myocardial infarction a promising role for circulating microRNAs**. Biomarkers.

[15] Dorval V, Nelson PT, Hebert SS (2013). **Circulating microRNAs in Alzheimer’s disease: the search for novel biomarkers**. Front Mol Neurosci.

[16] Deddens JC, Colijn JM, Oerlemans MI, Pasterkamp G, Chamuleau SA, Doevendans PA, Sluijter JP (2013). **Circulating microRNAs as novel biomarkers for the early diagnosis of acute coronary syndrome**. J Cardiovasc Transl Res.

[17] Ramshankar V, Krishnamurthy A (2013). **Lung cancer detection by screening - presenting circulating miRNAs as a promising next generation biomarker breakthrough**. Asian Pac J Cancer Prev.

[18] Dart DA, Waxman J, Bevan CL, Sita-Lumsden A (2013). **Circulating microRNAs as potential new biomarkers for prostate cancer**. Br J Cancer.

[19] Grasedieck S, Sorrentino A, Langer C, Buske C, Dohner H, Mertens D, Kuchenbauer F (2013). **Circulating microRNAs in hematological diseases: principles, challenges, and perspectives**. Blood.

[20] Redova M, Sana J, Slaby O (2013). **Circulating miRNAs as new blood-based biomarkers for solid cancers**. Future Oncol.

[21] Leidinger P, Backes C, Deutscher S, Schmitt K, Mueller SC, Frese K, Haas J, Ruprecht K, Paul F, Stahler C, Lang CJ, Meder B, Bartfai T, Meese E, Keller A (2013). **A blood based 12-miRNA signature of Alzheimer disease patients**. Genome Biol.

[22] Kumar P, Dezso Z, MacKenzie C, Oestreicher J, Agoulnik S, Byrne M, Bernier F, Yanagimachi M, Aoshima K, Oda Y (2013). **Circulating miRNA biomarkers for Alzheimer’s disease**. PLoS ONE.

[23] Taguchi YH, Murakami Y (2013). **Principal component analysis based feature extraction approach to identify circulating microRNA biomarkers**. PLoS ONE.

[24] Wehrens R, Franceschi P, Vrhovsek U, Mattivi F (2011). **Stability-based biomarker selection**. Anal Chim Acta.

[25] Vlachos IS, Kostoulas N, Vergoulis T, Georgakilas G, Reczko M, Maragkakis M, Paraskevopoulou MD, Prionidis K, Dalamagas T, Hatzigeorgiou AG (2012). **DIANA miRPath v.2.0: investigating the combinatorial effect of microRNAs in pathways**. Nucleic Acids Res.

[26] Maclellan SA, Lawson J, Baik J, Guillaud M, Poh CF, Garnis C (2012). **Differential expression of miRNAs in the serum of patients with high-risk oral lesions**. Cancer Med.

[27] Shen J, Wang A, Wang Q, Gurvich I, Siegel AB, Remotti H, Santella RM (2013). **Exploration of genome-wide circulating microRNA in hepatocellular carcinoma (HCC): MiR-483-5p as a potential biomarker**. Cancer Epidemiol Biomarkers Prev.

[28] Chan M, Liaw CS, Ji SM, Tan HH, Wong CY, Thike AA, Tan PH, Ho GH, Lee AS (2013). **Identification of circulating microRNA signatures for breast cancer detection**. Clin Cancer Res.

[29] Rommer A, Steinleitner K, Hackl H, Schneckenleithner C, Engelmann M, Scheideler M, Vlatkovic I, Kralovics R, Cerny-Reiterer S, Valent P, Sill H, Wieser R (2013). **Overexpression of primary microRNA 221/222 in acute myeloid leukemia**. BMC Cancer.

[30] Murakami Y, Toyoda H, Tanahashi T, Tanaka J, Kumada T, Yoshioka Y, Kosaka N, Ochiya T, Taguchi YH (2012). **Comprehensive miRNA expression analysis in peripheral blood can diagnose liver disease**. PLoS ONE.

[31] R Core Team (2013). R: A Language and Environment for Statistical Computing.

[32] Benjamini Y, Hochberg Y (1995). **Controlling the false discovery rate: a practical and powerful approach to multiple testing**. J R Stat Soc Series B (Methodological).

