# Supplementary material for: Universal disease biomarker: can a fixed set of blood microRNAs diagnose multiple diseases?
Source: BMC Res Notes. 2014 Aug 30;7:581. doi: 10.1186/1756-0500-7-581 (PMC4161864; doi:10.1186/1756-0500-7-581)
Supplement: Supplementary file 2 — Additional file 2: Supporting Figures. (PDF 198 KB) [file 13104_2013_3114_MOESM2_ESM.pdf]

Figure S1: Two dimensional embedding of miRNAs used for FE. Red triangles are selected miRNAs.

Alzheimer Disease

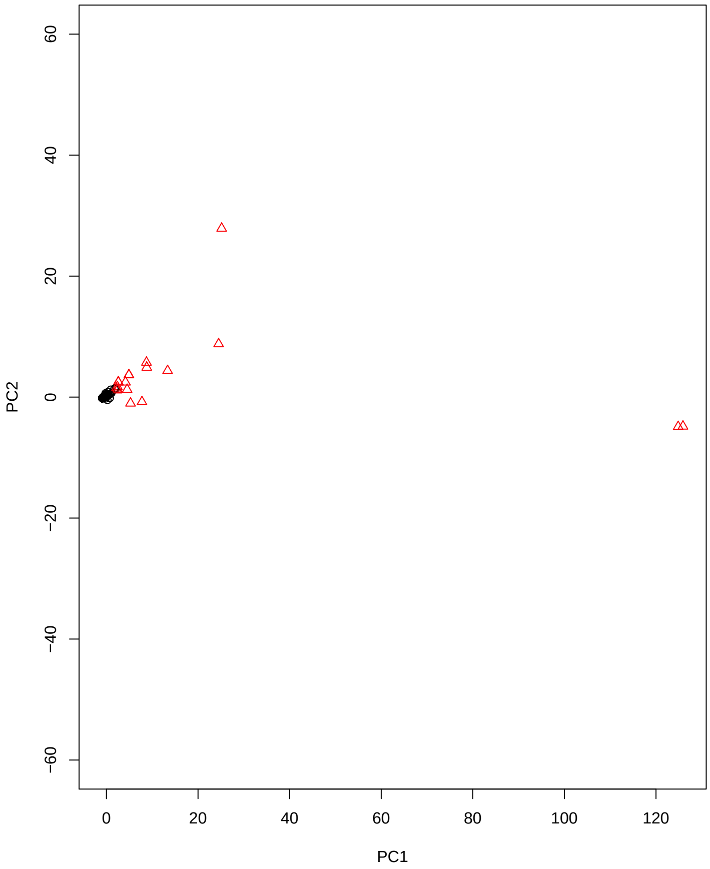

carcinoma

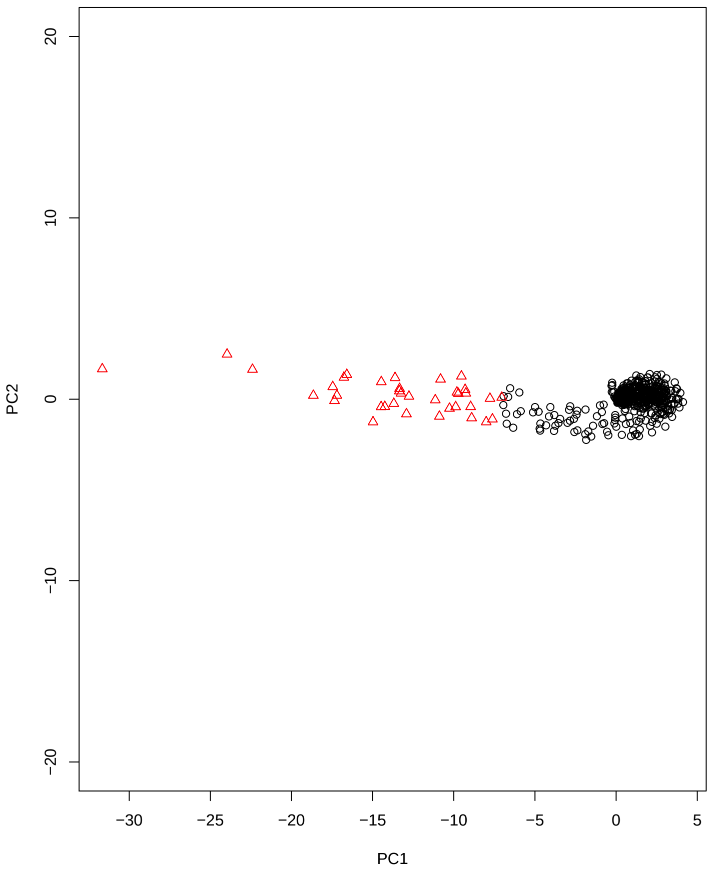

CAD

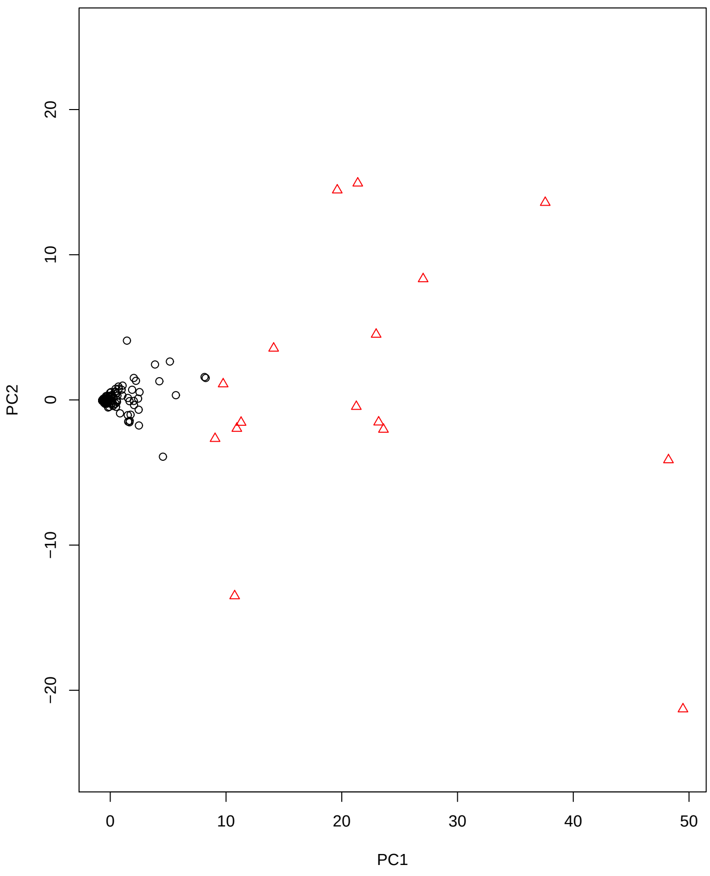

NPC

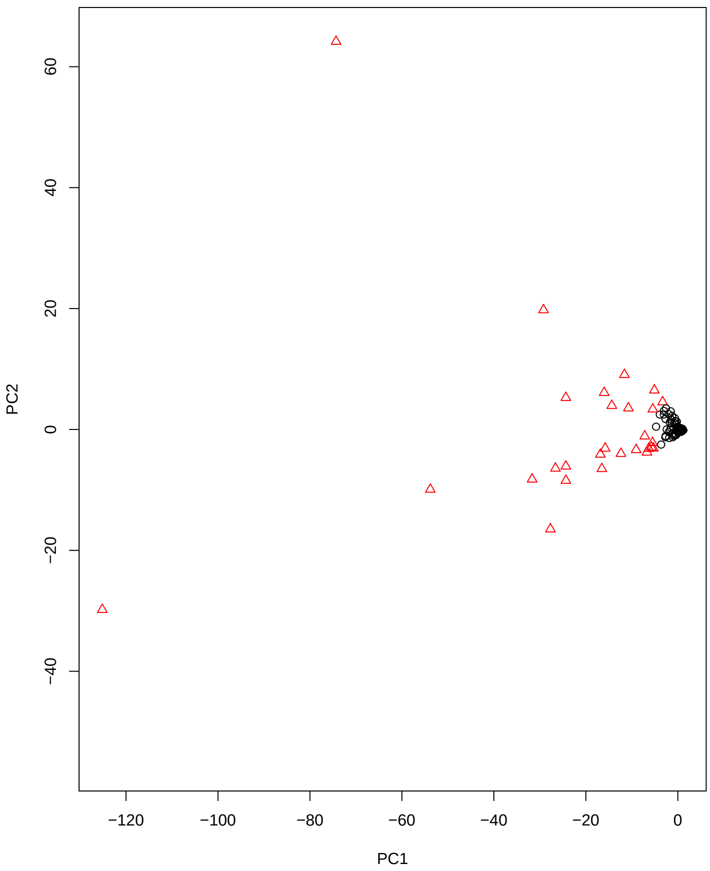
PC1
PC2
−120
−100
−80
−60
−40
−20
0
60
40
20
0
−20
−40

HCC

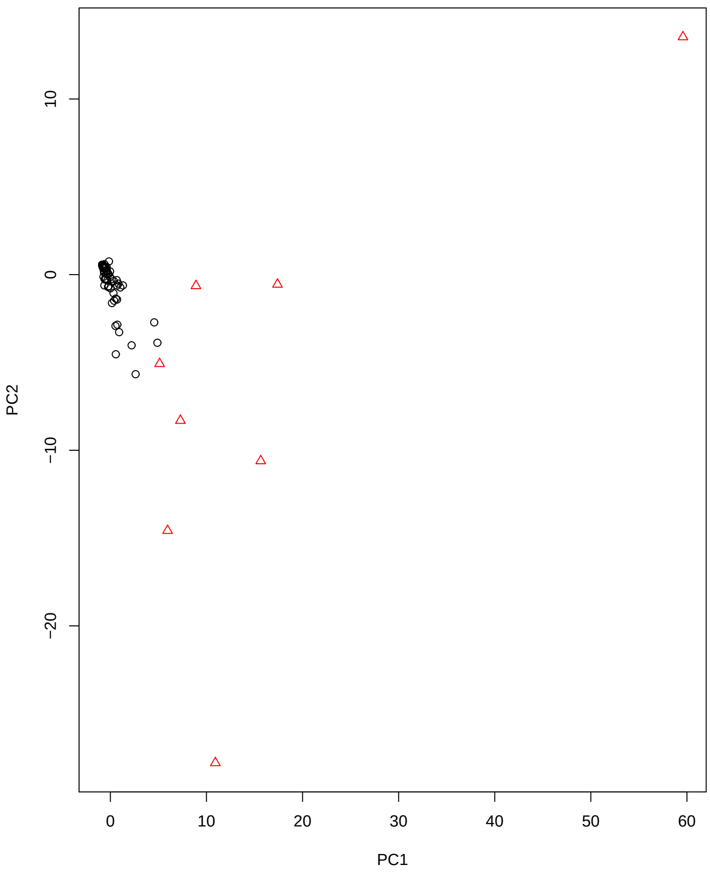
PC1
PC2
0
10
20
30
40
50
60
10
0
−10
−20

BC

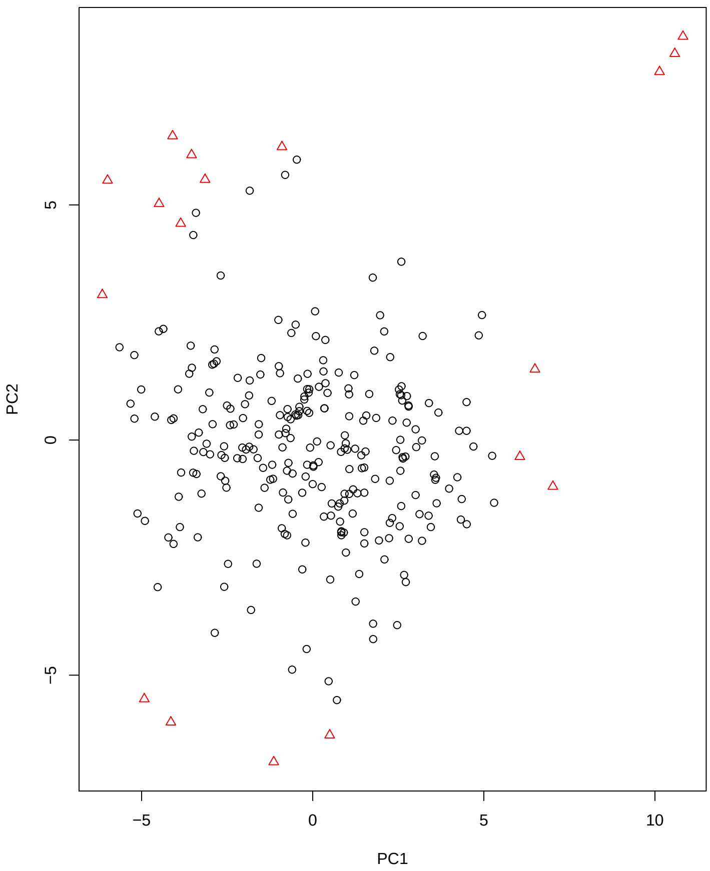

AML

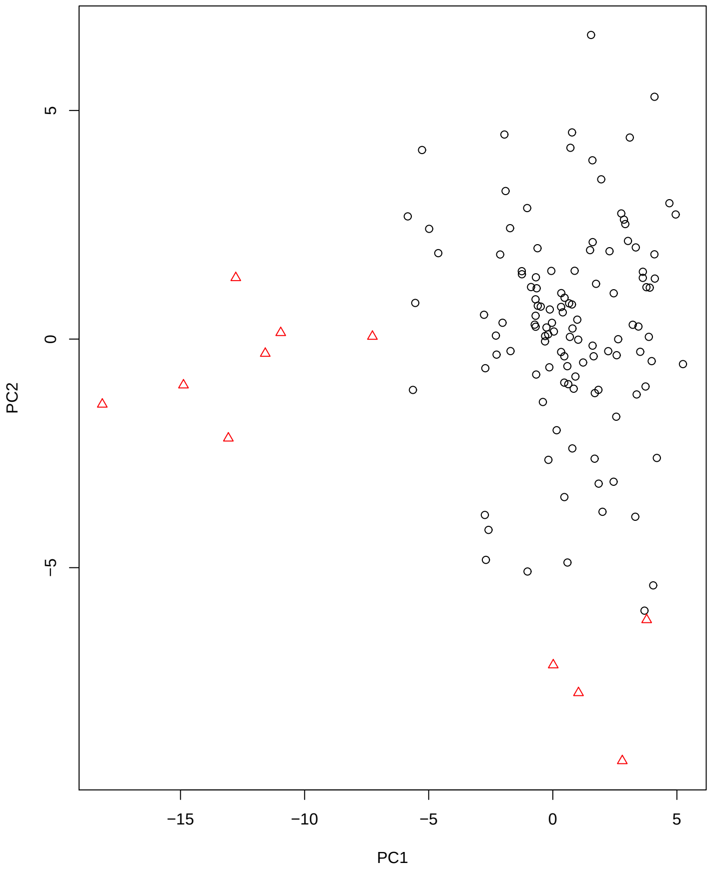

Figure S2 : Histogram of the number of selected miRNAs in FE and the scatter plot of the mean number of selected miRNAs between lasso and PCA based FE. The red solid line in the scatter plot indicates linear regression with assuming that intersect is zero (the slope is 1.11 and $P = 0.001$).

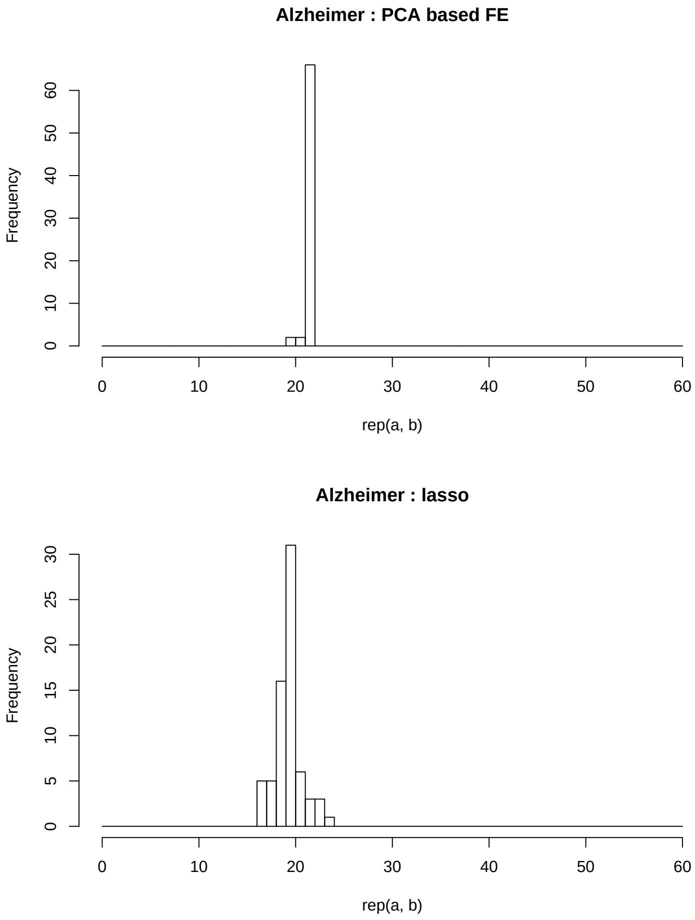

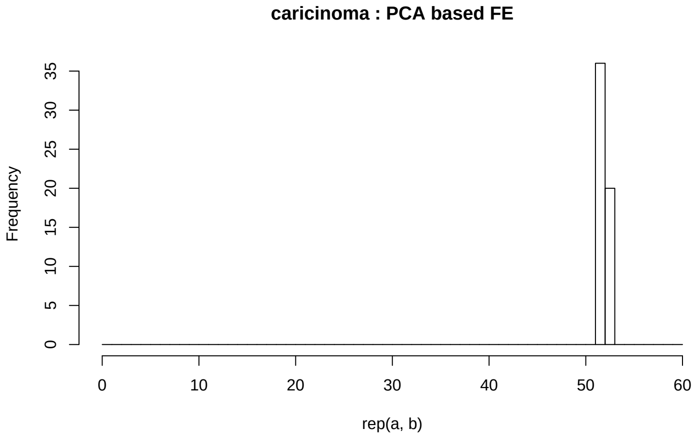
caricinoma : PCA based FE
Frequency
0
5
10
15
20
25
30
35
0
10
20
30
40
50
60
rep(a, b)

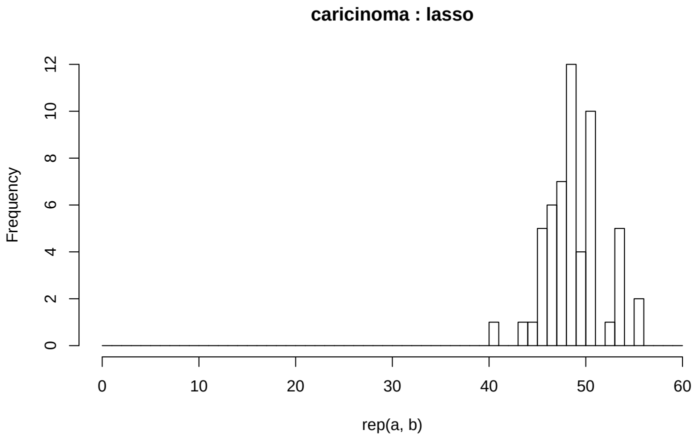
caricinoma : lasso
Frequency
0
2
4
6
8
10
12
0
10
20
30
40
50
60
rep(a, b)

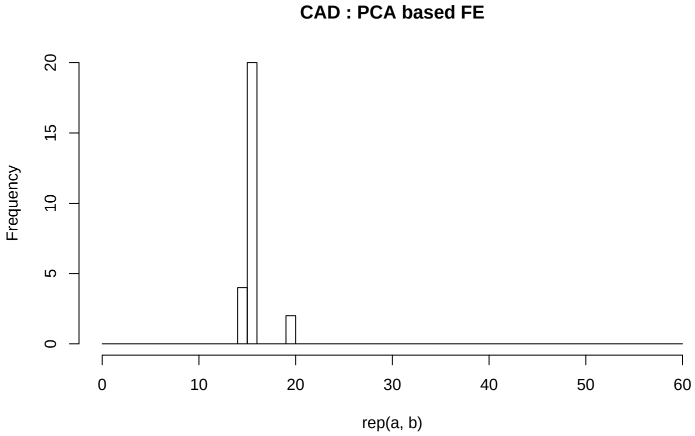
CAD : PCA based FE
Frequency
0
5
10
15
20
0
10
20
30
40
50
60
rep(a, b)

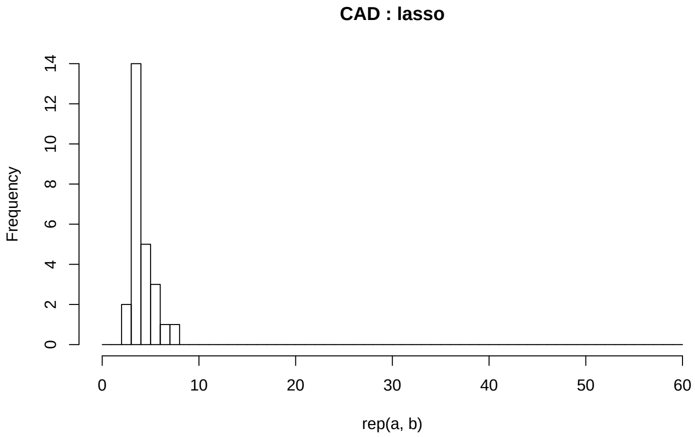
CAD : lasso
Frequency
0
2
4
6
8
10
12
14
0
10
20
30
40
50
60
rep(a, b)

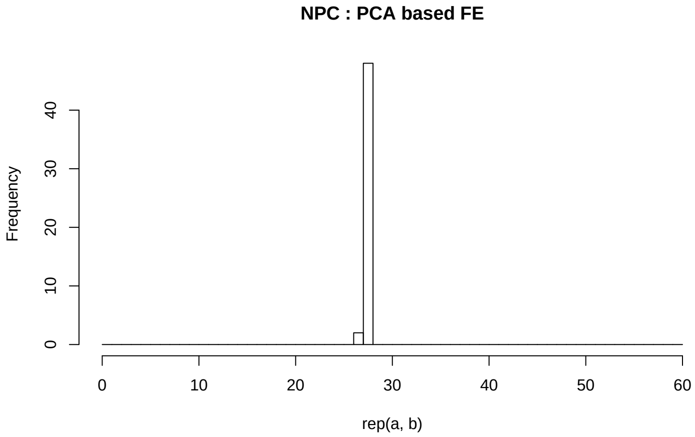
NPC : PCA based FE
Frequency
0 10 20 30 40
0 10 20 30 40 50 60
rep(a, b)

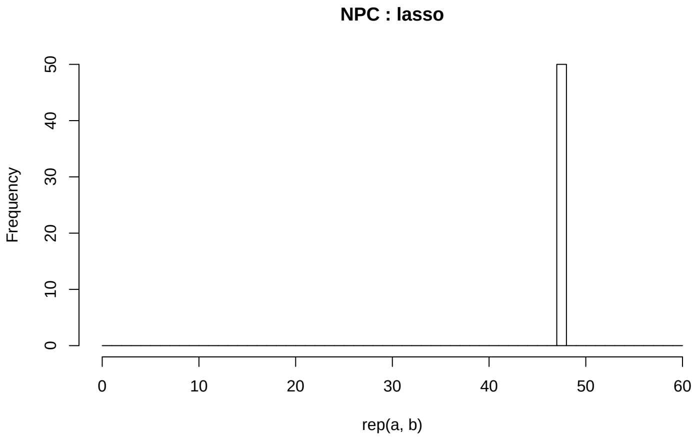
NPC : lasso
Frequency
0 10 20 30 40 50
0 10 20 30 40 50 60
rep(a, b)

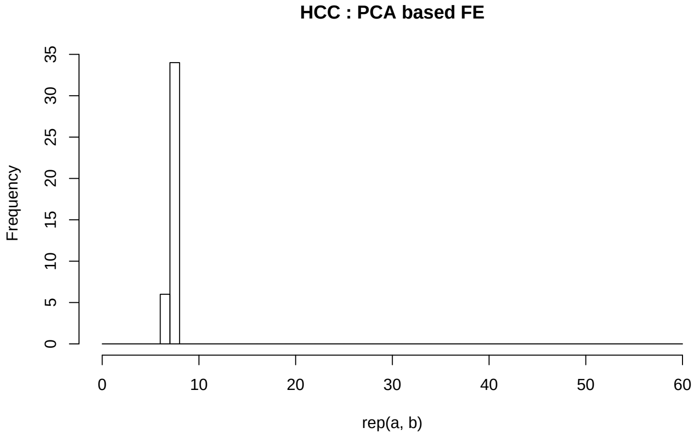
HCC : PCA based FE
Frequency
0 5 10 15 20 25 30 35
0 10 20 30 40 50 60
rep(a, b)

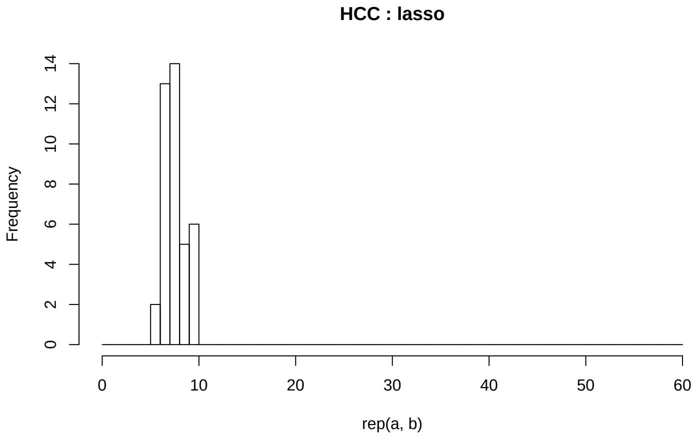
HCC : lasso
Frequency
0 2 4 6 8 10 12 14
0 10 20 30 40 50 60
rep(a, b)

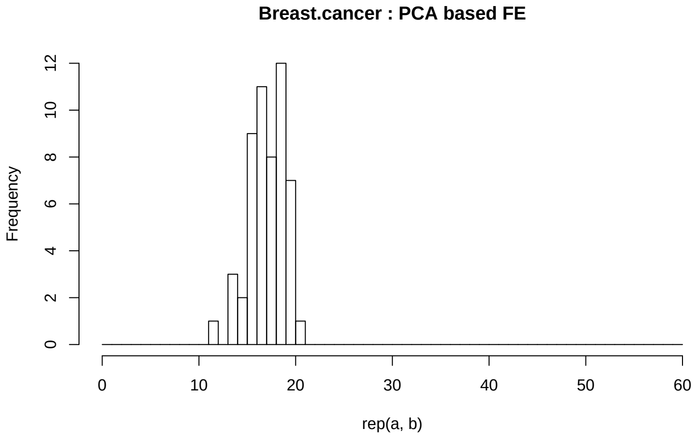
Breast.cancer : PCA based FE
Frequency
0 2 4 6 8 10 12
0 10 20 30 40 50 60
rep(a, b)

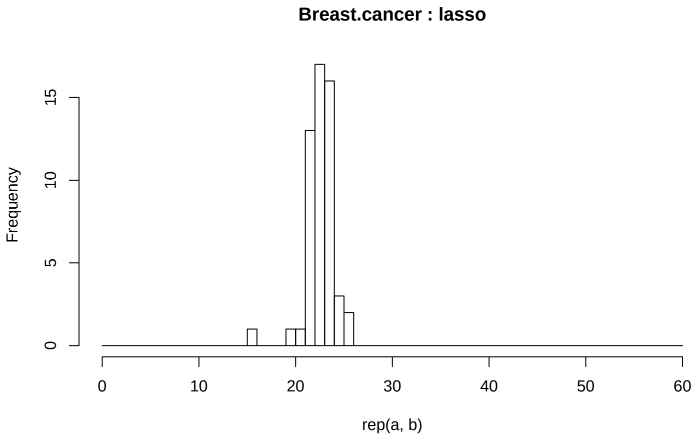
Breast.cancer : lasso
Frequency
0 5 10 15
0 10 20 30 40 50 60
rep(a, b)

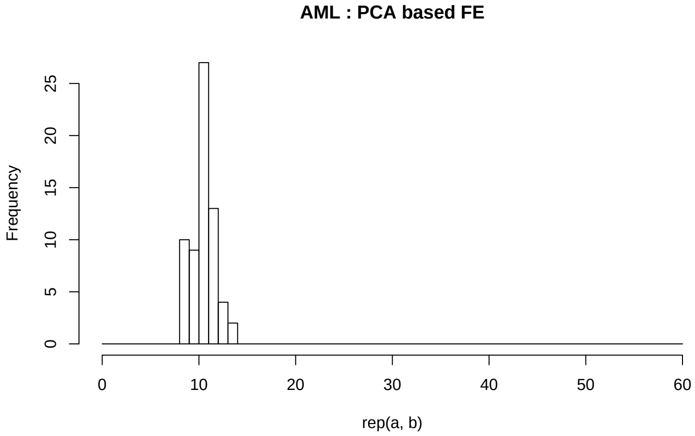
AML : PCA based FE
Frequency
0 5 10 15 20 25
0 10 20 30 40 50 60
rep(a, b)

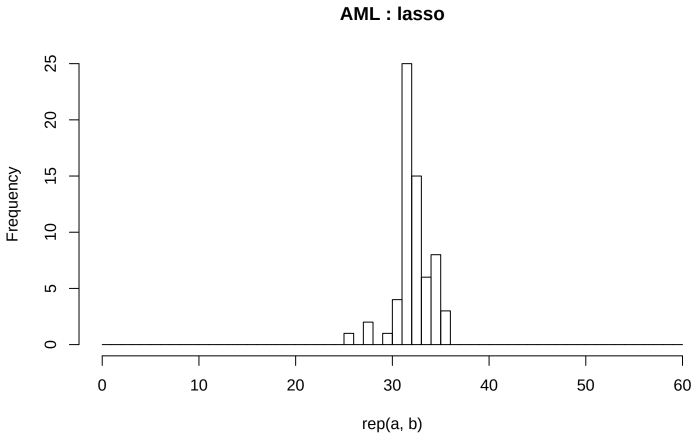
AML : lasso
Frequency
0 5 10 15 20 25
0 10 20 30 40 50 60
rep(a, b)

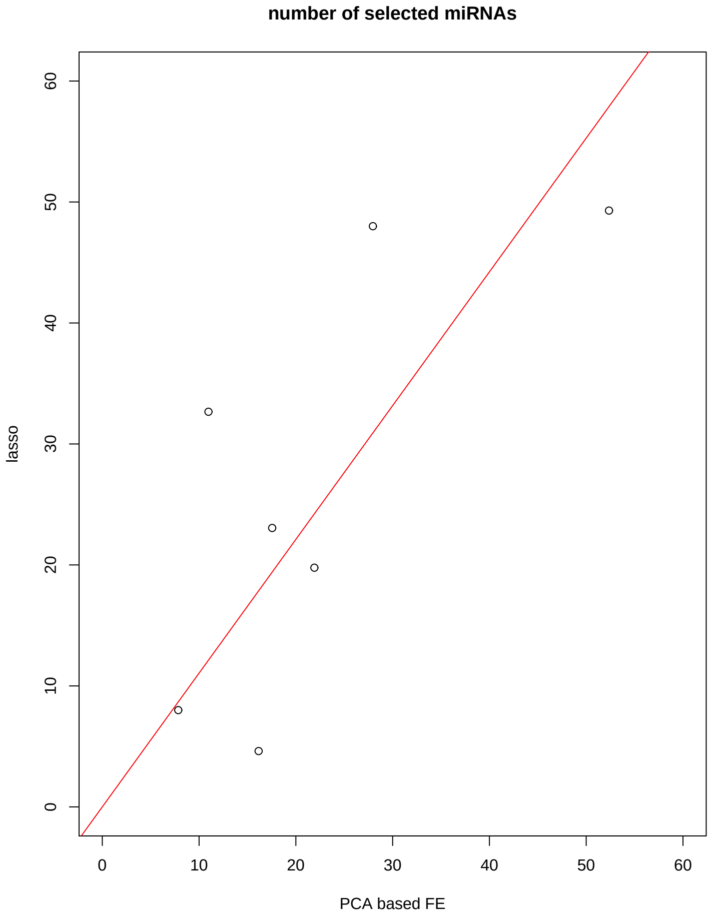
number of selected miRNAs
lasso
PCA based FE
0
10
20
30
40
50
60
