# Supplementary material for: Universal disease biomarker: can a fixed set of blood microRNAs diagnose multiple diseases?
Source: BMC Res Notes. 2014 Aug 30;7:581. doi: 10.1186/1756-0500-7-581 (PMC4161864; doi:10.1186/1756-0500-7-581)
Supplement: Supplementary file 3 — Additional file 3: Supporting Texts. (ZIP 17 KB) [file 13104_2013_3114_MOESM3_ESM.zip › Text_S1.pdf]

The representation of PCA-based unsupervised FE using singular value decomposition

Since singular value decompostion (SVD) is an alternative method of matrix diagonalization, it may be helpful if we clearly denote the relationship between PCA and SVD in our case. As has been explained in our paper,

$$\frac{1}{N}\mathbf{X}^T\mathbf{X}\boldsymbol{u}_k = \lambda_k \boldsymbol{u}_k, (k = 1, \cdots, M) \tag{1}$$

Multiplying $\boldsymbol{u}_{k'}^T$ to eq.(1) from left hand side, we get

$$\frac{1}{N}\boldsymbol{u}_{k'}^T\mathbf{X}^T\mathbf{X}\boldsymbol{u}_k = \boldsymbol{u}_{k'}^T\lambda_k \boldsymbol{u}_k, (k, k' = 1, \cdots, M)$$

Then we get

$$\frac{1}{N}\left(\mathbf{X}\boldsymbol{u}_{k'}\right)^T\mathbf{X}\boldsymbol{u}_k = \lambda_k\delta_{kk'}, (k, k' = 1, \cdots, M)$$

where $\delta_{kk'}$ is Kronecker delta. This can be rewritten as

$$V\mathbf{X}U = \Lambda \tag{2}$$

where

$$\begin{aligned}
V &= \begin{pmatrix} \boldsymbol{v}_1^T \\ \boldsymbol{v}_2^T \\ \cdots \\ \boldsymbol{v}_M^T \end{pmatrix} \\
\boldsymbol{v}_k &= \mathbf{X}\boldsymbol{u}_k \\
U &= (\boldsymbol{u}_1, \boldsymbol{u}_2, \cdots, \boldsymbol{u}_M) \\
\Lambda &= N\begin{pmatrix} \lambda_1 & 0 & & \cdots & 0 \\ 0 & \lambda_2 & 0 & \cdots & 0 \\ & & \cdots & & \\ 0 & 0 & & \cdots & \lambda_M \end{pmatrix}
\end{aligned}$$

Since $U^TU = V^TV = I$, multiplying $V^T(U^T)$ to eq.(2) from the left(right) hand side, we get

$$\mathbf{X} = V^T\Lambda U^T$$

that is SVD. In our PCA-based unsupervised FE, X is replaced with X' where

$$\begin{aligned}
x'_{ij} &= x_{ij}, i \leq N' \leq N \\
&= 0, N' < i \leq N, \\
&\quad (j = 1, \cdots, M)
\end{aligned}$$

and x_{ij}s, $(i \leq N)$ have relatively larger PCSs, $x_{ik} \equiv \sum_j u_{kj}x_{ij}, k \leq K$. Since $v_{ki} = x_{ik}$, this corresponds to replace $\boldsymbol{v}_k = (v_{k1}, \cdots, v_{kN})$ with $\boldsymbol{v}'_k = (v_{k1}, \cdots, v_{kN'}, 0, \cdots, 0)$. However, since it does not satisfy, for example, $\boldsymbol{v}'_k \cdot \boldsymbol{v}'_{k'} = \delta_{kk'}$, any more, the whole computation must be repeated.
